# Chemical Constituents of the Bark of *Dipteryx alata* Vogel, an Active Species against *Bothrops jararacussu* Venom

**DOI:** 10.3390/molecules15118193

**Published:** 2010-11-12

**Authors:** Pilar Puebla, Yoko Oshima-Franco, Luiz M. Franco, Marcio G. Dos Santos, Renata V. da Silva, Leandro Rubem-Mauro, Arturo San Feliciano

**Affiliations:** 1 Department of Pharmaceutical Chemistry, Faculty of Pharmacy, Campus "Miguel de Unamuno", Salamanca University, E-37007 Salamanca, Spain; 2 Sorocaba University, UNISO, Rodovia Raposo Tavares km 92.5, 18023-000, Sorocaba, SP, Brazil; E-Mail: franco@prof.uniso.br (Y. O.-F.); 3 Methodist University of Piracicaba, Rodovia do Açúcar km 156, 13400-911, Piracicaba, SP, Brazil; E-Mail: lenof@terra.com.br (L.M.F.); 4 Federal University of Tocantins, Av. NS 15 ALC NO 14, 109 Norte, 77001-090, Porto Nacional, TO, Brazil; E-Mail: galdino@uft.edu.br (M.G.D.S.)

**Keywords:** *Dipteryx alata*, Leguminosae-Papilionoideae, isoflavone, *Bothrops jararacussu*, snake venom, neutralization

## Abstract

The effect of four sub-extracts prepared from the lyophilized hydroalcoholic bark of *Dipteryx alata* (Leguminosae-Papilionoideae) dissolved in a methanol-water (80:20) mixture through a liquid-liquid partition procedure has been investigated against the neuromuscular blockade of the venom of the snake *Bothrops jararacussu*. The active CH_2_Cl_2_ sub-extract has been extensively analyzed for its chemical constituents, resulting in the isolation of four lupane-type triterpenoids: lupeol (**1**), lupenone (**2**), 28-hydroxylup-20(29)-en-3-one (**3**), betulin (**4**), nine isoflavonoids: 8-*O*-methylretusin (**5**), 7-hydroxy-5,6,4’-trimethoxyisoflavone (**6**), afrormosin (**8**), 7-hydroxy-8,3’,4’-trimethoxyisoflavone (**9**), 7,3’-dihydroxy-8,4’-dimethoxyisoflavone (**10**), odoratin (**11**), 7,8,3’-trihydroxy-4’-methoxyisoflavone (**13**), 7,8,3’-trihydroxy-6,4’-dimethoxyisoflavone (**15**), dipteryxin (**17**), one chalcone: isoliquiritigenin (**7**), one aurone: sulfuretin (**14**) and three phenolic compounds: vanillic acid (**12**), vanillin (**16**), and protocatechuic acid (**18**). Their chemical structures were elucidated on the basis of spectroscopic analysis, including HRMS, 1D- and 2D-NMR techniques.

## 1. Introduction

*Dipteryx alata* Vogel, belongs to the Leguminosae-Papilionoideae family, and is a native species of the Brazilian Savanna, found principally in Minas Gerais, Goiás, Federal District and Mato Grosso. This species is known popularly as “baru” in Minas Gerais, “barujo” and “combaru” in Mato Grosso, and “cumaru” in other states. The plant has great economic potential because of its multiple uses. The fruit, rich in proteins, fibres and unsaturated fatty acids, is used as human and animal food, and also in cosmetic formulations [1,2,3]. Traditionally, the local inhabitants use the oil extracted from the almond to treat high fevers, and also for snakebites.

No extensive phytochemical investigations on *Dipteryx alata* were found, and the only previous report on the composition of this plant described the isolation of three lupane triterpenoids [4]. Previously, we have described the activity of *D. alata* methanolic extract obtained by Soxhlet extraction against the neuromuscular blockade of *Bothrops jararacussu* venom [5] from the pharmacological point of view. Exploiting the potential of the lyophilized *D. alata* hydroalcoholic extract, a liquid-liquid partition procedure based on varying polarity was carried out. This paper describes the results of a new investigation of the baru bark against the neuromuscular blockade produced by the jararacuçu snake (*Bothrops jararacussu*). Baru bark sub-extracts were obtained using apolar to polar types of solvents (hexane, dichloromethane, ethyl acetate and methanol); the efficacy of each was evaluated against muscle tissue damage and the neuromuscular blocking effects of *Bothrops jararacussu* venom.

**Figure 1:**
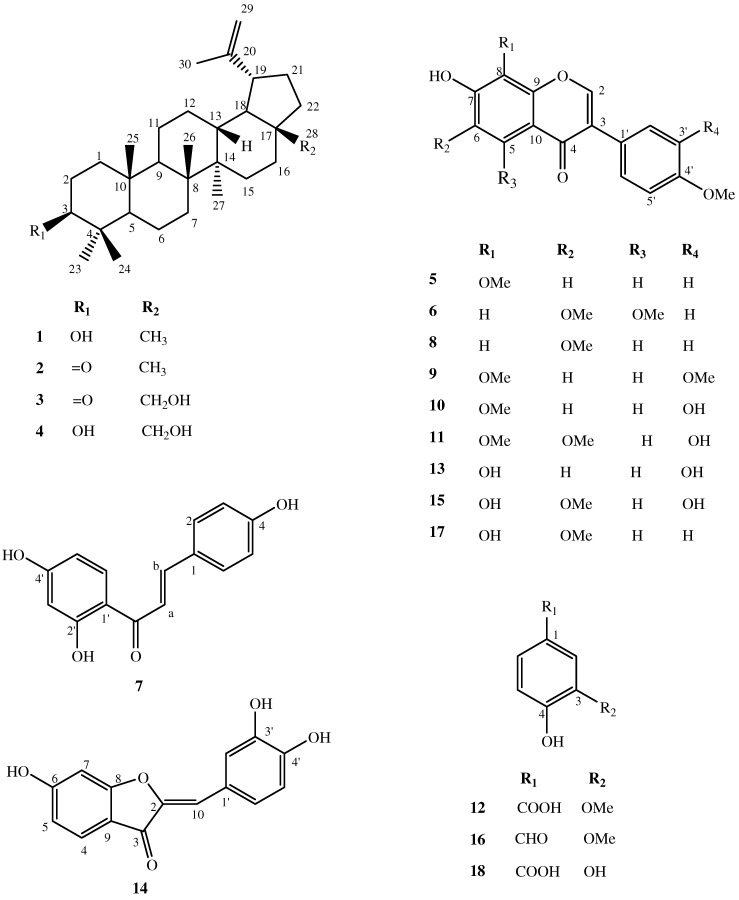
Chemical structures of compounds **1–18**.

The CH_2_Cl_2_ sub-extract was one of the most active. For this reason, we have examined the constituents of this fraction, which has led to the isolation of four lupane-type triterpenoids **1-4**, nine isoflavonoids **5, 6, 8-11, 13, 15, 17**, one chalcone **7**, one aurone **14** and three phenolic compounds **12**, **16** and **18** (Figure 1). All these compounds, with the exception of triterpenoids **1**, **2** and **4**, have been isolated from *D. alata* for the first time. The isoflavone **6** had been previously synthesized and isolated by acid hydrolysis of an isoflavone glycoside from other source, while it is being reported here as a natural aglycone. The isoflavone **13** had been isolated previously as an acetate derivative, and it is reported here as a natural product for the first time. Full spectroscopic data for the isolated isoflavonoids, including ^13^C-NMR assignments, previously not described in literature, are reported herein. 

## 2. Results and Discussion

The 70% EtOH extract of the bark of *Dipteryx alata* was lyophilized, dissolved in a methanol-water (80:20) mixture and sequentially fractioned with hexane, CH_2_Cl_2_ and EtOAc. The CH_2_Cl_2_-soluble portion of this extract was subjected to repeated column chromatography in silica gel, and Sephadex LH-20 to yield twelve fractions (F1–F12) that afforded 18 compounds (Figure 1).

The triterpenoids were readily identified as lupeol (**1**), lupenone (**2**), 28-hydroxylup-20(29)-en-3-one (**3**) and betulin (**4**) [4,6,7,8] by a GC-MS library search. Isoflavonoids were identified by comparison with literature data and descriptions as 8-*O*-methylretusin (7-hydroxy-8,4’-dimethoxyisoflavone) (**5**) [9,10], 7-hydroxy-5,6,4’-trimethoxyisoflavone (**6**) [11,12], afrormosin (7-hydroxy-6,4’-dimethoxyisoflavone) (**8**) [13], 7-hydroxy-8,3’,4’-trimethoxyisoflavone (**9**) [14], 7,3’-dihydroxy-8,4’-dimethoxyisoflavone (**10**) [14,15], odoratin (7,3’-dihydroxy-6,4’-dimethoxyisoflavone) (**11**) [16], 7,8,3’-trihydroxy-4’-methoxyisoflavone (**13**) [17], 7,8,3’-trihydroxy-6,4’-dimethoxy-isoflavone (**15**) [18], dipteryxin (7,8-dihydroxy-6,4’-dimethoxyisoflavone) (**17**) [15]. The chalcone was identified as isoliquiritigenin (4,2’,4’-trihydroxychalcone) (**7**) [19], the aurone as sulfuretin (6,3’,4’-trihydroxyaurone) (**14**) [20], and the phenolic compounds as vanillic acid (**12**) [21], vanillin (**16**)[21], and protocatechuic acid (**18**) [22]. Their chemical structures were determined on the basis of complete spectroscopic analysis, including HR-ESI-MS, 1D- and 2D-NMR techniques and comparison with data reported in the literature. 

Compound **6** gave a pseudomolecular ion peak at *m*/*z* 351.0847 [M + Na]^+^ by HR-ESI-MS consistent with the molecular formula C_18_H_16_O_6_Na. The ^1^H-NMR spectrum (Table 1) showed a singlet for H-2 at δ 7.80, characteristic of an isoflavone, another singlet at δ 6.78 corresponding to H-8 showed HMBC correlations with the signals of quaternary carbons at δ 153.8 (C-7), 154.6 (C-9) and 138.2 (C-6). Two doublets of double intensity at δ 6.96 and 7.47 confirmed the presence of a *p*-substituted ring B. Three *O*-methyl singlets at 3.84, 3.95 and 4.03 showed HMBC correlations with ^13^C signals at 159.4 (C-4’), 151.7 (C-5) and 138.2 (C-6) respectively, indicating the location of the *O*-methyl groups. The ^13^C-NMR (Table 2) data are in accordance with those reported for the aglycone obtained by acid hydrolysis of an isoflavonoid glycoside isolated from *Baphia bancoensis* [12], and also with a synthetic compound [11].

**Table 1:** ^1^H-NMR spectral data, δ in ppm, *J* in Hz, for compounds **5**, **6**, **8**, **9** and **10**.

H	5 (CDCl_3_)	6 (CDCl_3_)	8 (DMSO)	9 (DMSO)	10 (CD_3_OD)
**2**	8.00 s	7.80 s	8.29 s	8.43 s	8.16 s
**5**	7.95 d 8.9		7.42 s	7.71 d 8.9	7.77 d 8.9
**6**	7.05 d 8.9			7.01 d 8.9	6.96 d 8.9
**8**		6.78 s	6.93 s		
**2’**	7.48 d 8.8	7.47 d 8.7	7.50 d 8.9	7.16 d 1.8	7.02 br s
**3’**	6.97 d 8.8	6.96 d 8.7	6.94 d 8.9		
**5’**	6.97 d 8.8	6.96 d 8.7	6.94 d 8.9	6.98 d 8.4	6.97 br s
**6’**	7.48 d 8.8	7.47 d 8.7	7.50 d 8.9	7.11 dd 8.4;1.8	6.93 br s
**OMe**	3.82 s	3.84 s	3.77 s	3.76 s	3,.84 s
	(OMe-4’)	(OMe-4’)	(OMe-4’)	(OMe-3’)	(OMe-4’)
**OMe**	4.06 s	3.95 s	3.86 s	3.76 s	3.93 s
	(OMe-8)	(OMe-5)	(OMe-6)	(OMe-4’)	(OMe-8)
**OMe**		4.03 s		3.85 s	
		(OMe-6)		(OMe-8)	

**Table 2:** ^13^C-NMR spectral data, δ in ppm, for compounds **5**, **6**, **8**, **9** and **10**.

C	5 (CDCl_3_)	6 (CDCl_3_)	8 (DMSO)	9 (DMSO)	10 (CD_3_OD)
**2**	151.9	150.6	152.8	153.3	154.6
**3**	124.7	125.0	124.7	123.0	125.6
**4**	176.4	175.3	174.2	174.7	178.0
**5**	122.0	151.7	104.6	120.7	122.3
**6**	114.0	138.2	146.9	115.2	112.5
**7**	153.4	153.8	152.7	154.8	156.5
**8**	134.0	99.0	102.8	134.6	136,2
**9**	150.2	154.6	151.7	150.6	152.6
**10**	118.6	113.3	116.2	117.4	119.1
**1’**	124.0	124.1	122.6	124.4	126.1
**2’**	130.2	130.3	130.0	112.7	117.4
**3’**	113.9	113.8	113.5	148.6	147.4
**4’**	159.6	159.4	158.9	148.2	149.2
**5’**	113.9	113.8	113.5	111.5	116.5
**6’**	130.2	130.3	130.0	121.2	121.6
**OMe**	55.3 (OMe-4’)	55.3 (OMe-4’)	55.7(OMe-6)	55.5 (OMe-3’)	56.4 (OMe-4’)
**OMe**	61.8 (OMe-8)	61.7 (OMe-6)	55.1 (OMe-4’)	55.5(OMe-4’)	61.8 (OMe-8)
**OMe**		61.9 (OMe-5)		60.7(OMe-8)	

Compound **13** was isolated as a yellow amorphous solid. The ^1^H-NMR of **13** (Table 3) showed a singlet at 8.17 for H-2 typical of an isoflavone. The aromatic region of the spectrum displayed an A_2_B_2_-system (two doublets at δ 6.94 and 7.57, *J* = 8.7) characteristic for the A-ring in 7,8-dioxygenated isoflavones, and signals for three protons at δ 6.93, 6.96 and 7.03 of the disubstituted B-ring. 

**Table 3:** ^1^H-NMR spectral data, δ in ppm, *J* in Hz, for compounds **11**, **13**, **13a**, **15** and **17**.

H	11 (CDCl_3_)	13 (CD_3_OD)	13a (CDCl_3_)	15 (CD_3_OD)	17 (CD_3_OD)
**2**	7.91 s	8.17 s	8.21 s	8.10 s	8.09 s
**5**	7.3 s	7.57 d 8.7	8.03 d 8.9	7.11 s	7.07 s
**6**		6.94 d 8.7	7.30 d 8.9		
**8**	6.96 s				
**2’**	7.12 s	7.03 s	7.40 br s	7.02 s	7.35 d 8.6
**3’**					6.86 d 8.6
**5’**	6.90 d 9.0	6.96 s	7.01 br s	6.90 d 1.5	6.86 d 8.6
**6’**	7.10 d 9.0	6.93 s	7.45 br s	6.90 d 1.5	7.35 d 8.6
**OMe**	3.90 s	3.80 s	3,86 s	3.83 s	3.71 s
	(OMe-4’)		(OMe-4’)	(OMe-4’)	
**OMe**	4.0 3H s			3.90 3H s	3.80 s
	(OMe-6)			(OMe-6)	
**OMe**					
**Ac**			2.29, 2.36, 2.42		

From the above data and the ^13^C-NMR signals (Table 4), this compound could be identified as 7,8,3’-trihydroxy-4’-methoxyisoflavone. To confirm this structure, compound **13** was acetylated. The ^1^H-NMR spectrum of the acetyl derivative, **13a**, showed three singlets at δ 2.29, 2.36 and 2.42 corresponding to the tri-*O*-acetyl derivative. Other ^1^H-NMR data for **13a** (Table 3) were in good agreement with those reported for an isoflavone isolated as acetate from *Xanthocercis zambesiaca* [17].

**Table 4:** ^13^C-NMR spectral data, δ in ppm, for compounds **11**, **13**, **15** and **17**.

C	11 (CDCl_3_)	13 (CD_3_OD)	15 (CD_3_OD)	17 (CD_3_OD)
**2**	152.3	154.6	154.2	154.2
**3**	125.2	125.3	124.8	124.8
**4**	175.6	178.5	178.0	178.0
**5**	104.7	115.4	96.2	96.2
**6**	145.5	112.6	148.4	148.5
**7**	152.4	151.0	134.6	134.6
**8**	102.6	134.1	141.6	141.7
**9**	151.7	147.4	144.3	144,5
**10**	117.7	118.8	117.3	117,2
**1’**	124.0	126.3	126.3	125.6
**2’**	115.2	117.3	117.4	131.4
**3’**	145.4	147.8	147.2	114.7
**4’**	146.5	149.2	148.9	160.9
**5’**	110.6	117.4	112.4	114.7
**6’**	121.0	121.6	121.6	131.4
**OMe**	55.9 (OMe-4’)	56.4	56.3 (OMe-4’)	55.6 (OMe-4’)
**OMe**	56.4 (OMe-6)		56.5 (OMe-6)	56.2 (OMe-6)

### 2.1. Anti-venom Assays

The protective activity of the four sub-extracts (hexane, dichloromethane, ethyl acetate and residual methanol) of the EtOH/H_2_O extract from *Dipteryx alata*, against the neuromuscular blockade of jararacuçu venom (*Bothrops jararacussu,* Bjssu venom), was confirmed by biological assays.

### 2.2. Mouse phrenic nerve-diaphragm (PND) preparation

The phrenic nerve-diaphragm muscle was obtained from mice previously anesthetized with halothane and sacrificed by exsanguination. The diaphragm was removed and mounted as described by Bülbring [23]. PND preparations were allowed to stabilize for at least 20 min before addition of one of the following solutions: Tyrode solution (control, n = 5); 40 µg/mL Bjssu (n = 6) venom, or by the mixture of venom plus 50 μg/mL of each extracts obtained by liquid partition [hexane, Hex (n = 4); dichloromethane, Dcm (n = 4); ethyl acetate, Eac (n = 5) or methanol, Met; n = 4] 30 min before addition to the neuromuscular preparation. The sub-extract concentration was chosen since did cause no effect on the basal response of preparation. 

**Figure 2:**
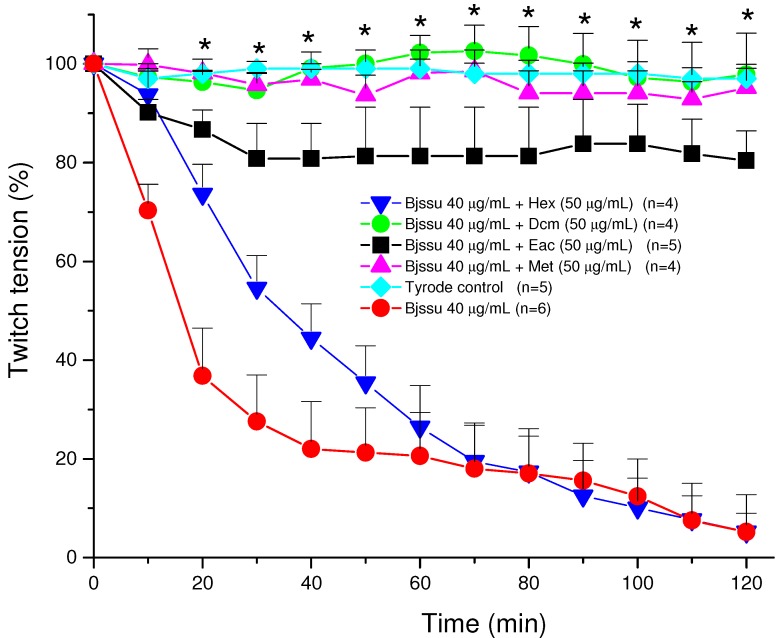
Pharmacological assays on mouse phrenic nerve-diaphragm (PND) preparations to determine the twitch response under electric indirect stimuli after incubation the preparation with 40 µg/mL *B. jararacussu* (Bjssu) venom plus *D. alata* bark extracts (50 μg/mL) using different sub-extracts (Hex, hexane; Dcm, dichloromethane; Eac, ethyl acetate; Met, methanol). Note methanol and dichloromethane extracts 100% prevented the neuromuscular blockade promoted by Bjssu venom alone. Each point represents the mean ± S.E.M. of the number of experiments (n) showed in the legend. *All points from this time interval (20 min) onwards were significantly different (p<0.05 for Dcm, Eac, and Met mixtures) from the venom.

The Figure 2 shows the pharmacological profile of the *in vitro* neutralization assays after mixing 50 μg/mL of each extract (Hex, Dcm, Eac, and Met) with Bjssu venom (40 µg/mL) prior to test. Note that hexane (Hex) did not protect (5.2% ± 3.7) against irreversible neuromuscular blockade induced by crude venom (5% ± 5.6). A fair protection was seen with the ethyl acetate sub-extract (80% ± 6), whereas a practically total protection was seen with dichloromethane (98% ± 8) and methanol (95% ± 4.7) sub-extracts (* p < 0.05 compared to venom). As it can be seen, the Dcm and Met extracts showed no significant difference in relation with Tyrode control.

These results represent an attempt at identifying the active compounds responsible by the protective action of *D. alata* against *Bothrops jararacussu* venom, since preliminary data obtained from thin layer chromatography comparison pointed out to phenolic compounds present in the *D. alata* hydroalcoholic extract [5].

According to the screening of some chromatography fractions of the Dcm sub-extract, the F7 fraction displayed the most efficacious protection against the venom effect (results not shown). Taken these results together, compounds **12** (vanillic acid), **13 **(3’,7,8-trihydroxy-4’-methoxyisoflavone) and **14 **(3’,4’,6-trihydroxyaurone), found in that fraction, could be the main responsible substances for the observed protection. Additionally, it can be stated that the triterpenoids are not responsible for the anti-*Bothrops jararacussu* venom activity, because they are also abundant in the inactive hexane sub-extract.

The mechanism by which these phytochemicals could neutralize the crude venom is yet unclear and further studies should be done. In fact, for investigating the mechanism of action or the pharmacological response, each isolated compound should be individually assayed against the venom, a condition not always possible due to the small isolated amounts. Besides, isolated compounds not always preserve the same pharmacological efficacy as that seen with total extract, as already related by elsewhere using the same experimental model [24,25].

## 3. Experimental

### 3.1. General

IR spectra were obtained on a Nicolet Impact 410 spectrophotometer. ^1^H-NMR (200 and 400 MHz), and ^13^C-NMR (100 and 50 MHz) spectra were recorded on Bruker AC 200 (200 MHz) and Bruker DRX 400 (400 MHz) spectrometers, with TMS as an internal reference, δ given in ppm and *J* in Hz. 2D-NMR spectra were measured with Brüker DRX 400 spectrometer. HR ESI MS analysis, a VG-TS250 mass spectrometer (70 eV) was used. GC-MS analysis was performed on a Hewlett Packard 5890 series II, chromatograph apparatus equipped with Hewlett-Packard 5971 mass spectrometer operating in the EIMS-mode at 70 eV and Supelco SPB-1 column (12 m × 0.20 mm, with 0.33 μm film thickness) with helium (10 Psi), as carrier gas at a flow rate of 1 mL/min. The GC oven temperature was kept at 90 °C for 5 minutes and programmed to 250 °C with a gradient of 2 °C /min and maintained for 10 min at 250 °C, Wiley 275 as research library. Silica gel (230–400 mesh) for column chromatography and GF254 for TLC were obtained from Merck KGaA, 64271 Darmstadt, Germany. Sephadex LH-20 was obtained from Fluka, BioChemika Switzerland. 

### 3.2. Statistical analysis

The results were expressed as the mean ± S.E.M. Student’s *t*-test was used for statistical comparison of the data and the significance level was set at 5%.

### 3.3. Plant material

The bark of *Dipteryx alata* was collected in Pedro Afonso in the Tocantins Estate of Brazil, in August of 2007. The plant was identified by Dr. Roseli B. Torres from “Núcleo de Pesquisa e Desenvolvimento do Jardin Botânico”, Institute of Agronomy of Campinas. A voucher specimen: IAC 50629 was deposited in the Institute of Agronomy of Campinas (Brazil).

### 3.4. Venom

*B. jararacussu venom (Bjssu)* was kindly donated by Prof. Dr. José Carlos Cogo from Universidade do Vale do Paraiba-UNIVAP (São José dos Campos, São Paulo state, Brazil).

### 3.5. Animals

Male Swiss white mice (26­32 g) were supplied by Anilab: Animais de Laboratório (Paulínia, S.P., Brazil). The animals were housed at 25 ± 3 °C on a 12 h light/dark cycle and had access to food and water *ad libitum*. This project (protocol n^o^ A012/CEP/2006) was approved by the institutional Committee for Ethics in Research of Vale do Paraiba University (UNIVAP), and the experiments were carried out according to the guidelines established by the Brazilian College for Animal Experimentation (COBEA).

### 3.6. Extraction and isolation

The air-dried bark of *D. alata* (1.269 Kg) was extracted with 70% EtOH at room temperature for 2 hours, and the solution was evaporated *in vacuum* to give a residue (50 g). The residue was dissolved in a 80:20 methanol-water mixture and partitioned successively with the corresponding solvents to give a hexane (1.5 g), CH_2_Cl_2_ (18 g), EtOAc (3.7 g), and MeOH residue (21 g). The hexane fraction was crystallized to yield 1.4 g of compound **1**. The CH_2_Cl_2 _fraction was subjected to silica gel flash column chromatography and eluted with hexane-EtOAc (9:1 to EtOAc) to give 12 fractions, that were further successively flash-chromatographed on silica gel and purified by Sephadex LH-20 column chromatography, eluted with hexane-CH_2_Cl_2_-MeOH–H_2_O (2:2:1) to yield 18 compounds: **2** (216 mg), **1** (1,004 mg), **3** (180 mg), **4** (523 mg), **5** (49 mg), **6 **(20 mg), **7** (12 mg), **8** (9 mg), **9** (25 mg), **10 **(41 mg), **11** (28 mg), **12** (8 mg), **13** (19 mg), **14** (6 mg), **15** (70 mg), **16** (12 mg), **17** (15 mg), **18** (200 mg).

*8-O-Methylretusin* (**5**): Pale yellow amorphous solid. IR (film) cm^−1^: 3,232, 1,606, 1,511, 1,449, 1,381. ^1^H-NMR data see Table 1. ^13^C-NMR data see Table 2. HR-ESI-MS *m*/*z*: 321.0734 [M + Na]^+^ (Calcd for C_17_H_14_O_5_Na, 321.0733).

*7-Hydroxy-5,6,4’-trimethoxyisoflavone* (**6**): Yellow amorphous solid. IR (film) cm^−1^: 3,394, 1,609, 1,511, 1,464, 1,379. ^1^H-NMR data see Table 1. ^13^C-NMR data see Table 2. HR-ESI-MS *m*/*z*: 351.0847 [M + Na]^+^ (Calcd for C_18_H_16_O_6_Na, 351.0839).

*Afrormosin* (**8**): White amorphous solid.^ 1^H-NMR data see Table 1. ^13^C-NMR data see Table 3.

*7-Hydroxy-8,3’,4’-trimethoxyisoflavone* (**9**): Colourless amorphous solid. IR (KBr) cm^−1^: 3,400, 1,598, 1,517, 1,448. ^1^H-NMR data see Table 1. ^13^C-NMR data see Table 2.

*7,3’-Dihydroxy-8,4’-dimethoxyisoflavone* (**10**): Yellow amorphous solid. IR (KBr) cm^−1^: 3,429, 1,624, 1,590, 1,513, 1,256. ^1^H-NMR data see Table 1. ^13^C-NMR data see Table 2. HR-ESI-MS *m*/*z*: 337.0681 [M + Na]^+^ (Calcd for C_17_H_14_O_6_Na, 337.0684).

*Odoratin* (**11**): Colourless amorphous solid. IR (film) cm^−1^: 3,409, 1,617, 1,598, 1,513, 1,328. ^1^H-NMR data see Table 3. ^13^C-NMR data see Table 4. HR-ESI-MS *m*/*z*: 337.0678 [M + Na]^+^ (Calcd for C_17_H_14_O_6_Na, 337.0682).

*7,8,3’-Trihydroxy-4’-methoxyisoflavone* (**13**): Yellow amorphous solid. IR (KBr) cm^−1^: 3,429, 1,619, 1,604, 1,290, 1,124. ^1^H-NMR data see Table 3. ^13^C-NMR data see Table 4.

*7,8,3’-Triacetoxy-4’-methoxyisoflavone* (**13a**): The compound **13a **was obtained from compound **13** (19 mg) after acetylation with Ac_2_O/Py, as brown amorphous solid (15 mg). ^1^H-NMR data see Table 3. 

*7,8,3’-Trihydroxy-6,4’-dimethoxyisoflavone* (**15**): Yellow amorphous solid. IR (KBr) cm^−1^: 3,410, 1,604, 1,513, 1,475, 1,130. ^1^H-NMR data see Table 3. ^13^C-NMR data see Table 4. HR-ESI-MS *m*/*z*: 337.0617 [M + Na]^+^ (Calcd for C_17_H_14_O_7_Na, 353.0631).

*Dipteryxin* (**17**): Yellow amorphous solid. IR (KBr) cm^−1^: 3,420, 1,621, 1,513, 1,465, 1,380. ^1^H-NMR data see Table 3. ^13^C-NMR data see Table 4. HR-ESI-MS *m*/*z*: 337.0677 [M + Na]^+^ (Calcd for C_17_H_14_O_6_Na, 337.0682).

## 4. Conclusions

In summary, four sub-extracts from *D. alata* have been examined for their protective activity against the neuromuscular blockade caused by *Bothrops jararacussu* venom. The active CH_2_Cl_2_ extract has been extensively analyzed for its chemical constituents, resulting in the isolation of four lupane-type triterpenoids, nine isoflavonoids, one chalcone, one aurone and three phenolic compounds. On the basis of the present study, further pharmacological evaluation of each isolated compound will be need to ascertain which of them are responsible for the activity, as well as for establishing the mechanism of action.

## References

[1] Takemoto E., Okada I.A., Garbelotti M.L., Tavares M., Aued-Pimentel S. (2001). Chemical composition of seeds and oil of baru (*Dipteryx alata* Vog.) native from Pirenópolis, State of Goiás, Brazil. Rev. Inst. Adolfo Lutz.

[2] Vera R., Soares Soares M., Veloso Naves R., Barboza de Souza E.R., Fernandes E.P., Caliari M., Mozena Leandro W. (2009). Chemical characteristics of baru almonds (*Dipteryx alata* Vog.) from the Savannah of Goiás, Brazil. Rev. Bras. Frutic. Jaboticabal.

[3] Fernandes D.C., Freitas J.B., Czeder L.P., Naves M.M.V. (2010). Nutritional composition and protein value of the baru (*Dipteryx alata* Vog.) almond from the Brazilian Savanna. J. Sci. Food Agric..

[4] Coelho Kaplan M.A., Gottlieb O.R., Gilbert B., Salignac de Souza Guimaraes I., Taveira Magalhaes M. (1966). Química de Leguminosas Brasileiras. Derivados do Lupeol em *Dipteryx alata*. An. Acad. Bras. Cienc..

[5] Nazato V.S., Rubem-Mauro L., Vieira N.A.G., dos Santos Rocha-Junior D., Glauzer Silva M., Santos Lopes P., Dal-Belo C.A., Cogo J.C., dos Santos M.G., da Cruz-Höfling M.A., Oshima-Franco Y. (2010). *In Vitro* Antiophidian Properties of *Dipteryx alata* Vogel Bark Extracts. Molecules.

[6] Mutai C., Abatis D., Vagias C., Moreau D., Roussakis C., Roussis V. (2004). Cytotoxic lupane-type triterpenoids from *Acacia mellifera*. Phytochemistry.

[7] Tinto W.F., Blair L.C., Azzan Alli, Reynolds W.F., McLean S. (1992). Lupane triterpenoids of *Salacia cordata*. J. Nat. Prod..

[8] Shehla I., Iqbal A.M., Mohtasheemul H.M.S., Waseemuddin A. (2007). Two triterpenes lupenone and lupeol isolated and identified from *Tamarindus indica* Linn. Pak. J. Pharm. Sci..

[9] Agrawal P.K. (1989). Carbon-13 NMR of Flavonoids.

[10] Jurd L., Stevens K., Manners G. (1972). Isoflavones of the heartwood of *Dalbergia retusa*. Phytochemistry.

[11] Horie T., Shibata K., Yamashita K., Fujii K., Tsukayama M., Ohtsuru Y. (1998). Studies of the Selective *O*-Alkylation and Dealkylation of Flavonoids. XXIV. A Convenient Method for Synthesizing 6- and 8-Methoxylated 5,7-Dihydroisoflavones. Chem. Pharm. Bull..

[12] Yao-Kouassi P., Magid A.A., Richard B., Martinez A., Jacquier M.J., Caron C., Le Magrex Debar E., Gangloff S.C., Coffy A. A., Zèches-Hanrot M. (2008). Isoflavonoid Glycosides from the Roots of *Baphia bancoensis*. J. Nat. Prod..

[13] Gong T., Wang D.-X., Chen R.-Y., Liu P., Yu D.-Q. (2009). Novel Benzil and Isoflavone Derivatives from *Millettia dielsiana*. Planta Med..

[14] Harper S.H., Shirley D.B., Taylor D.A. (1976). Isoflavones from *Xanthocercis zambesiaca*. Phytochemistry.

[15] Hayashi T., Thomson R.H. (1974). Isoflavones from *Dipteryx odorata*. Phytochemistry.

[16] Januário A.H., Lourenço M.V., Domézio L.A., Pietro R.C.L.R., Castilho M.S., Tomazela D.M., da Silva M.F.G.F., Vieira P.C., Fernandes J.B., Castro França S. (2005). Isolation and Structure Determination of Bioactive Isoflavones from Callus Culture of *Dipteryx odorata*. Chem. Pharm. Bull..

[17] Bezuidenhout S.C., Bezuidenhout B.C.B., Ferreira D. (1988). α-Hydroxydihydrochalcones and related 1,3-diarylpropan-2-ones from *Xanthocercis zambesiaca*. Phytochemistry.

[18] Socorro M.P., Pinto A.C., Kaiser C.R.Z. (2003). New Isoflavonoids from *Dipteryx odorata*. Naturforsch.

[19] Ma C.-J., Li G.-S., Zhang D.-L., Liu K., Fan X. (2005). One step isolation and purification of liquiritigenin and isoliquiritigenin from *Glycyrrhiza uralensis* Risch. Using high-speed counter-current chromatography. J. Chromatogr. A.

[20] Júnior G.M.V., Sousa C.M., Cavalheiro A.J., Lago J.H.G., Chaves M.H. (2008). Phenolic Derivatives from Fruits of *Dipteryx lacunifera* DUCKE and Evaluation of Their Antiradical Activities. Helv. Chim. Acta.

[21] Lee C.-K., Lu C.-K., Kuo Y.-H., Chen J.-Z., Sun G.-Z. (2004). New Prenylated Flavones from the Roots of *Ficus beecheyana*. J. Chin. Chem. Soc..

[22] Kang W.-Y., Li G.-H., Hao X.-J. (2003). Two New Triterpenes from *Neonauclea sessilifolia*. Acta Bot. Sin..

[23] Bülbring E. (1946). Observation on the isolated phrenic nerve diaphragm preparation of the rat. Br. J. Pharmacol..

[24] Cintra-Francischinelli M., Silva M.G., Andréo-Filho N., Gerenutti M., Cintra A.C.O., Giglio J.R., Leite G.B., Cruz-Höfling M.A., Rodrigues-Simioni L., Oshima-Franco Y.  (2008). Antibothropic action of *Casearia sylvestris* Sw.(Flacourtiaceae) extracts. Phytother. Res..

[25] Melo R.S., Farrapo N.M., Rocha-Junior D.S., Silva M.G., Cogo J.C., Dal Belo C.A., Rodrigues-Simioni L., Groppo F.C., Oshima-Franco Y. (2009). Flavonoids: Biosynthesis, Biological Effects and Dietary Sources.

